# Supplementary material for: Significant and Various Effects of ML329-Induced MITF Suppression in the Melanoma Cell Line
Source: Cancers (Basel). 2024 Jan 7;16(2):263. doi: 10.3390/cancers16020263 (PMC10814414; doi:10.3390/cancers16020263)
Supplement: Supplementary file 1 [file cancers-16-00263-s001.zip › cancers-2785186-supplementary.pdf]

Supplementary File
Table S1

KRAS	Taqman	TGCCCTACATCTTATTTCCTCAG	CCTACTGTCGCTAATGGATTGG	/56-FAM/AGGTGGTGG/ZEN/CTGATGCTTTGAACA/3IABkFQ/	NM_033360	6-6
SOX2	Taqman	GTACAACTCCATGACCAGCTC	CTTGACCACGAACCCAT	/56-FAM/CACCTACAG/ZEN/CATGTCCTACTCGCA/3IABkFQ/	NM_003106	1-1
MITF	Taqman	CTCACCATCAGCAACTCCTG	GATTGTCCTTTTTCTGCCTCTC	/56-FAM/AGCTCACAG/ZEN/CGTGTATTTTTCCCACA/3IABkFQ/	NM_000248	11-12a
HIF1a	Taqman	CCGTCATCTGTTAGCACCAT	GCTCACCATCAGTTATTTACGTG	/56-FAM/TCTAGACCA/ZEN/CCGGCATCCAGAAGT/3IABkFQ/	NM_010431	2-3
STAT3	Taqman	TGCTTCCCTGATTGTGACTG	AGGCATTTGGCATCTGACAG	/56-FAM/AGCTGCACC/ZEN/TGATCACCTTTGAGAC/3IABkFQ/	NM_213662	14-16
PGC1a	Taqman	GAGTCTGTTATGGAGTGACATCG	TGTCTGTATCCAAGTCGTTCAC	/56-FAM/ACCAGCCTC/ZEN/TTTGCCCAGATCTTC/3IABkFQ/	NM_013261	1-2

Supplemental Methods

2D and 3D cell culture of 4 MM cell lines

Five MM cells were each cultured in 2D culture dishes at 37°C in HG-DMEM culture medium supplemented with 8 mg/L d-biotin, 4 mg/L calcium pantothenate, 100 U/mL penicillin, 100 μg/mL streptomycin (b.p. HG-DMEM), 10 % CS and methylcellulose (Methocel A4M) until reaching approximately 90 % confluence. They were then divided into conventional 2D cultures and 3D spheroid cultures. The 2D cultured MM cells were then further maintained with medium changes daily for 7 days. Alternatively, for generating 3D spheroids, after washing with phosphate buffered saline (PBS), the cells were detached by treatment with 0.25 % Trypsin/EDTA and resuspended in the culture medium and then 28 μL of the medium containing approximately 20,000 cells was placed into each well of a drop culture plate (# HDP1385, Sigma-Aldrich) (3D/Day 0) as described previously [10, 51]. Thereafter, half of the culture medium was replaced with a fresh medium in each well daily until Day 7 [10, 51].

Extracellular flux assays in various MM cell lines

The day before the assay, the sensor cartridge was hydrated by ddH2O overnight at 37°C in a non-CO2 incubator and switched to the XF calibrant solution one hour before the assay. Twenty-four hours before the assay, various 2D cultured cells including WM226-4, SK-mel-24, A375, and A375DT cells that were treated or not treated with ML329 were seeded into the Seahorse ordinary 96-well plate at 20,000 cells per well and cultured at 37°C.

For the modified mitostress assay, one hour before the assay, the plates were washed and incubated in Seahorse XF DMEM assay medium (pH 7.4, Agilent Technologies, #103575-100) supplemented with 5.5 mM glucose, 2.0 mM L-glutamine, and 1.0 mM sodium pyruvate at 37°C in a non-CO2 incubator. After that, basal oxygen consumption rate (OCR) and extracellular acidification rate (ECAR) were simultaneously determined using a Seahorse XFe96 bioanalyzer and the samples were further analyzed after supplementation with oligomycin (final concentration of 2.0 μM), carbonyl cyanide p-trifluoromethoxyphenylhydrazone (FCCP, final concentration of 5.0 μM), rotenone and antimycin A (R/A, final concentration of 1.0 μM), and 2-deoxyglucose (2DG, final concentration of 10 mM final). For the glycolytic stress assay, the plates were replaced with Seahorse XF DMEM assay medium (pH 7.4) supplemented with 2.0 mM L-glutamine one hour before the assay and ECAR values were determined using a Seahorse XFe96 bioanalyzer at baseline. The samples were further analyzed with sequential injection of glucose (final concentration of 5.5 mM: low glucose condition or final concentration of 11 mM: high glucose condition), oligomycin (final concentration of 2.0 μM), and 2DG (final concentration of 50 mM).

After completion of the assays, the buffer was removed and cells remaining in the wells were lysed with 10 μL of Cell-Lytic buffer (#C3228, Sigma-Aldrich) and protein concentration was measured by the BCA protein assay (TaKaRa BIO, Shiga, Japan). Values of OCR and ECAR were normalized for the protein amount.

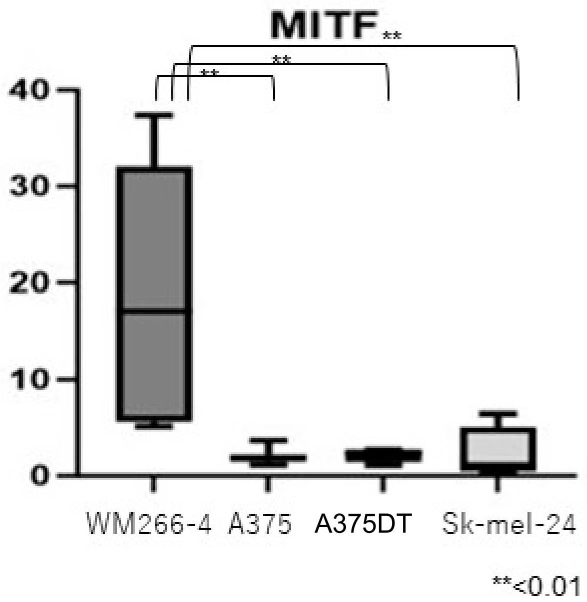

MITF
**
**
**
40
30
20
10
0
WM266-4
A375
A375DT
Sk-mel-24
**<0.01
